# Effect of Racking Systems Versus Enriched Terraria on Fecal Glucocorticoid Metabolites in Four Species of Captive Snakes: A Pilot Study

**DOI:** 10.3390/ani16101485

**Published:** 2026-05-12

**Authors:** Sergi Olvera-Maneu, Marçal Regidor, Paula Serres-Corral, Albert Martínez-Silvestre, Manel López-Béjar

**Affiliations:** 1 Department of Veterinary Medicine, University of Nicosia School of Veterinary Medicine, 2414 Nicosia, Cyprus; 2 Department of Animal Health and Anatomy, Universitat Autònoma de Barcelona, 08193 Bellaterra, Spainpaula.serres@uab.cat (P.S.-C.); manel.lopez.bejar@uab.cat (M.L.-B.); 3 Catalonian Reptiles and Amphibians Rescue Center, 08783 Masquefa, Spain; crarc@amasquefa.com

**Keywords:** reptiles, corticosterone, non-invasive sampling, HPA axis, HPI axis, environmental enrichment, animal welfare, stress

## Abstract

Fecal glucocorticoid metabolites (FGM) are widely used to assess hypothalamic–pituitary–adrenal (HPA) activity in vertebrates, but their applicability to reptiles, and more specifically to snakes, remains poorly explored. The objective of the present study was to determine how changes in housing conditions, from a standard racking system to enriched terraria, affect fecal glucocorticoid metabolite concentrations as a proxy potentially related to endocrine activity and stress response. A total of 58 fecal samples were collected across three different phases from 12 snakes representing four species: *Boa constrictor* (BC), *Lampropeltis polyzona* (LP), *Pantherophis guttatus* (PG), and *Python regius* (PR). FGM concentrations ranged from 54.9 to 832.2 ng/g, with a mean value of 298.4 ± 171.6 ng/g (mean ± SD). Data showed marked inter-individual variability, while within-individual concentrations remained relatively stable across experimental phases. PG exhibited the highest FGM concentrations during the study, with LP showing intermediate levels and PR and BC the lowest. The results showed that housing conditions did not have a significant effect on FGM concentrations in the studied population. In conclusion, the present study showed that fecal hormone analysis provides an integrated measure of glucocorticoid metabolites concentrations over time, potentially reflecting physiological differences among snake species.

## Simple Summary

This study evaluated whether fecal samples could effectively measure the stress response in snakes experiencing changes in housing conditions. Fecal glucocorticoid metabolites were quantified by using an enzyme immunoassay. Statistical analysis was performed to assess differences across housing types and species. No significant variation in hormone levels was observed between basic racking systems and enriched terraria. However, differences between species were observed. These results indicate that fecal hormone analysis could be a practical method to detect physiological differences among snake species, providing a cumulative measure of glucocorticoid metabolites over time.

## 1. Introduction

Over the past few decades, there has been a significant growth in the global ownership of exotic animals, with snakes being no exception [1]. In addition to being pets, snakes are also kept in captivity in various other settings, such as zoos, research and breeding facilities, and rescue centers. In these diverse environments the so-called “racking system” has emerged as one of the main housing systems [1]. Despite its practical advantages, this system also presents some potential problems such as, for example, limited space or restricted opportunities to express normal behaviors, among others [2,3]. All these elements are considered potential causes of long-term stress, and therefore a direct impact on the overall welfare status of the affected animals [2,3,4].

Given the potential for chronic stress to negatively affect the welfare of captive animals, investigating physiological responses to different housing conditions could help to generate evidence-based husbandry recommendations [4]. Among the most studied aspects of this physiological response is the activation of the hypothalamic–pituitary–adrenal (HPA) axis in mammals, or hypothalamic–pituitary–interrenal (HPI) axis in some reptile species [5,6]. As in other vertebrates, the reptilian hypothalamus releases corticotropin-releasing hormone, which in turn stimulates the pituitary gland to secrete adrenocorticotropic hormone (ACTH) [7]. Circulating ACTH stimulates the interrenal cells to synthesize and release glucocorticoids (mainly corticosterone in reptiles), which subsequently act on target organs and tissues [7]. In line with this, recent advances in stress physiology, driven by the importance of stress response for welfare and conservation, have focused on alternative approaches to measure glucocorticoids [7,8,9]. While blood has traditionally been used, practical and ethical concerns have encouraged the study of other minimally invasive matrices [9]. Among these options, measuring glucocorticoid metabolites in reptile feces has become a potentially useful alternative to blood sampling [7]. Different studies in some reptile species have shown how fecal glucocorticoid metabolites (FGM) respond to ACTH or exogenous corticosterone administration, correlating with blood levels, and therefore suggesting its reliability in measuring the HPI axis activity [10,11,12]. However, the use of FGM as a potential physiological indicator in snakes needs further investigation, as existing studies have focused predominantly on invasive plasma sampling, with non-invasive fecal measurement in snake-specific studies remaining particularly scarce [7,13,14,15,16,17].

The objective of the present study was to determine how changes in housing conditions, from a standard racking system to enriched terraria, affect fecal glucocorticoid metabolite concentrations in captive snakes, using FGM as a proxy for endocrine activity and chronic stress response. We hypothesized that housing conditions would significantly influence FGM concentrations, and that fecal hormone analysis would provide a non-invasive measure of HPA/HPI axis activity.

## 2. Materials and Methods

### 2.1. Study Subjects & Ethics

A total of 12 snakes (7 females and 5 males) were included in the study. The individuals belonged to four different species: *Boa constrictor* (BC, n = 2), *Lampropeltis polyzona* (LP, n = 2), *Pantherophis guttatus* (PG, n = 4), and *Python regius* (PR, n = 4). The animals belonged to an established group of snakes housed at the wildlife rehabilitation center CRARC (Catalonian Reptiles and Amphibians Rescue Center; Av. del Maresme, 45, 08783 Masquefa, Barcelona, Spain), either surrendered by private owners or confiscated from illegal keepers. All individuals had been housed at the rescue center for a minimum of six months prior to the start of the study. The exact age of the individuals was unknown upon arrival; age class (adult or juvenile) was, however, determined for all individuals. A detailed description of the animals (species, sex, age class and weight) can be seen in Table 1. All animals were in good health condition during the study. In accordance with institutional policy, ethical approval was not required for this study, given that data collection was restricted to non-invasive fecal sampling and no experimental procedures were performed beyond the transfer of animals to enriched terraria.

### 2.2. Study Design

The study was developed in three different phases (P1, P2, P3): P1 was carried out from 27 December 2021 to 25 January 2022; P2 from January 25 to 25 February 2022; and P3 from February 25 to 24 March 2022. During P1, snakes were individually housed in their usual housing conditions, in containers proportional to their size, using the racking system. In each container, cardboard sheets were used as substrate, and they were only provided with a water bowl. During P2, snakes were moved to new enriched habitats with dry leaves, tree branches for climbing, hiding spaces, bigger water bowls and also increased space (see Table 1). Consistent with the rescue center’s conditions, no additional lighting or heating was used throughout the study, with animals maintained under ambient temperatures ranging from 28 °C to 31 °C and a natural 12 h light:dark photoperiod. Finally, in P3 animals were moved to their original housing system (same as P1). The snakes were fed with frozen-thawed rodents every 2 weeks, according to the feeding protocol established in the rescue center. The containers and terraria were checked twice daily (once in the morning and once in the afternoon). Fecal samples were collected manually from the terraria without handling the animals. Samples were placed in individually labeled airtight plastic bags and stored at −18 °C until further analysis. The total number of samples per period is presented in Table 1.

### 2.3. Fecal Hormone Extraction and FGM Analysis

**Figure 1:**
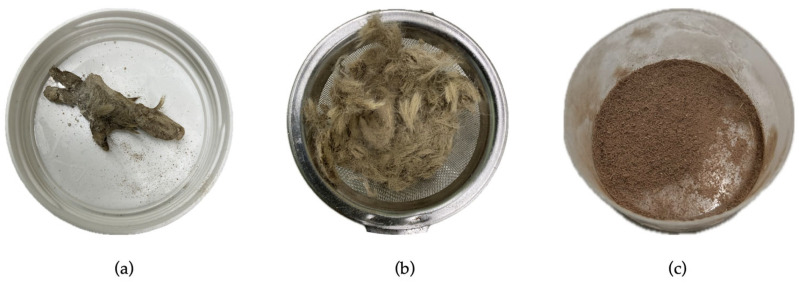
Fecal sample processing stages. (**a**) Sample appearance before trituration. (**b**) Intermediate stage, showing the substantial amount of rodent fur typically present in snake feces retained in the sieve with a mesh size smaller than 1 mm. (**c**) Final stage of the process, resulting in a homogenized sample with minimal residual fur and undigested material.

Samples were dried in a drying oven under constant airflow for over 36 h at 60 °C (Heraeus model T6; Kendro^®^ Laboratory Products, Langenselbold, Germany). Due to the high content of undigested rodent fur in the feces, conventional milling with a ball mill or similar mechanical machine was not performed [14]. Instead, samples were manually triturated using a mortar and passed through a sieve with a mesh size of 0.5 mm. This method retained most of the fur on the sieve, preventing excessive contamination of the triturated sample. Remaining fur that passed through the sieve was carefully removed with a slightly moistened small paintbrush, resulting in a nearly fur-free sample. A representation of the procedure is presented in Figure 1. From each triturated sample, 300 mg was weighed for hormone extraction following the protocol established by Palme et al. [18]. When insufficient sample mass was available, due to the high proportion of undigested prey material (fur, bones, etc.) typical of snake feces, the maximum amount possible was used maintaining the proportions established by the previously cited protocol. Briefly, 1 mL of 80% methanol (methanol reagent grade 99.9%; Scharlab, S.L., Sentmenat, Spain) was added per 100 mg of sample (1:100 proportion), mixed and vortexed (Vortex Mixer S0200-230 V-EU; Labnet International Inc., Edison, NJ, USA) for 30 min, and then centrifuged (Hermle Z300K; Hermle^®^ Labortechnik, Wehingen, Germany) at 5000 rpm (1750× *g*) for 15 min. When possible, 0.75 mL of the supernatant was collected in duplicate and stored at −20 °C until further analysis.

For the FGM quantification, a commercial enzyme immunoassay (EIA) kit was used (Corticosterone ELISA KIT; Neogen^®^ Corporation, Ayr, UK). This EIA kit presents cross-reactivity with deoxycorticosterone (38.0%), 6-hydrocorticosterone (19.0%), progesterone (5.1%), tetrahydrocorticosterone (2.7%), prednisolone (1.5%), cortisol (1.1%), pregnenolone (0.85%), 11-epicorticosterone (0.78%), cortisone (0.27%), 21-desoxycortisol (0.24%), d-aldosterone (0.13%), testosterone (0.12%), 17α-hydroprogesterone (0.12%), prednisone (0.10%), dexamethasone (0.03%), cholesterol (<0.01%), estradiol (<0.01%) and estriol (<0.01%). The corticosterone EIA was analytically validated using snake fecal extracts by verifying precision, specificity, accuracy, and sensitivity [9,18,19]. The tests were performed by using a constituted pool created with 50 μL from all included samples. The precision was assessed by calculating the coefficient of variation (CV) from all duplicated samples. For the specificity of the test, it was assessed by calculating the linearity of the dilution using 1:1, 1:2, 1:4 and 1:6 dilutions of the pool with the EIA buffer. Accuracy was assessed through the spike-and-recovery test, calculated by adding to 100, 75 and 25 μL of the pool, volumes of 25, 75 and 100 μL of three standard corticosterone concentrations provided by the kit (0.5, 1 and 2 ng/mL).

### 2.4. Statistical Analysis

Data analysis was performed using R software (Version 4.3.0; R Foundation for Statistical Computing, Vienna, Austria) and graphical representation was generated using GraphPad Prism (Version 9.5.1; Graph Pad Software Inc., San Diego, CA, USA). The level of statistical significance was established at *p* < 0.05 for all performed tests. The assumption of normality for the dependent variable (FGM) was evaluated using the Shapiro–Wilk test. Since the original data were non-normal, it was square-root-transformed. Phases P1 and P3 were grouped together under the variable housing (racking system) due to identical housing conditions, which was confirmed upon preliminary statistical testing showing no significant differences in FGM concentrations between both phases. A Linear Mixed Model (LMM), using the *lme4* package (Version 1.1.36) [20], was performed to evaluate variations in FGM, establishing species (BC, LP, PG, PR) and housing (racking system and enriched terraria) variables as fixed effects. The individuals were included as a random effect. The variables sex and age were excluded from the final model because of unbalanced representation across species, which prevented proper estimation of sex and age effects in the general model. Post hoc pairwise comparisons were applied only to significant main effects using Tukey’s adjustment with the *emmeans* package (Version 1.11.2) [21].

## 3. Results and Discussion

### 3.1. Analytical Validation of the EIA

Analytical validation is necessary before establishing reliable interpretations, especially when using less conventional matrices such as feces [9,18,19]. Based on this, the analytical validation of the EIA yielded the following results. The intra-assay coefficient of variation was 5.25%. The specificity test showed a correlation between the expected and observed FGM concentrations of 98.26% (r = 0.99; *p* < 0.05). The results of the spike-and-recovery test to measure accuracy presented a mean recovery percentage of 115.68% ± 19.71% (mean ± SD). Finally, sensitivity of the EIA for FGM was 0.11 ng/mL. The standard deviation obtained in the spike-and-recovery test indicates the presence of matrix interference, a frequent methodological challenge when analyzing complex biological matrices like feces. Coextracted compounds could interfere with assay performance and reduce absolute accuracy. Consequently, while the validated EIA is precise and sensitive enough to detect relative fluctuations in FGM, the absolute concentrations should be interpreted in accordance with the objective [22]. Future studies should aim to also biologically validate this assay, which would considerably strengthen the physiological interpretation of FGM concentrations in snakes, a group for which this type of validation remains scarce in the literature.

### 3.2. Quantification of Fecal Glucocorticoid Metabolite Concentrations

Fecal glucocorticoid metabolite concentrations ranged from 54.9 to 832.2 ng/g, with a mean value of 298.4 ± 171.6 ng/g (mean ± SD). Data showed marked inter-individual variability, while intra-individual concentrations remained relatively stable across experimental phases. Specifically, the mean within-subject coefficient of variation was 8.1%, indicating a low degree of individual fluctuation throughout the study.

The selected linear mixed effect model included the housing conditions and species as fixed effects and the individual as a random effect to account for repeated measurements of FGM. Sex and age, as stated before, were excluded due to unbalanced representation in the total sample, thus avoiding the introduction of singular fits and unreliable parameter estimates [23]. Housing conditions (racking system vs. enriched terrarium) did not have a significant effect on FGM concentrations (χ^2^ = 0.08; df = 1; *p* = 0.77). The absence of a significant effect of housing on FGM concentrations should be interpreted cautiously, as this outcome was likely influenced by several methodological and physiological factors rather than indicating that the habitat change had no physiological impact. First, the relatively small sample size, together with considerable inter-individual variation, reduced statistical power. Consequently, these results should not be interpreted as evidence that the environmental changes did not improve welfare or potentially reduce stress. Behavioral studies have indicated that environmental enrichment and more complex housing conditions can benefit snakes. For example, Hoehfurtner et al. [2] reported positive welfare effects of enrichment in *Pantherophis guttatus*. Similarly, Warwick et al. [1] found that snakes housed in larger enclosures exhibited a broader range of natural behaviors. In line with these findings, Hollandt et al. [24] documented an increase in natural behaviors and a reduction in abnormal ones when *Python regius* were moved from racking systems to more naturalistic terraria. Another factor that may have limited the detection of changes in HPI axis activity is the temporal dissociation between plasma glucocorticoid secretion and fecal metabolite excretion. Fecal glucocorticoid metabolites do not reflect acute endocrine activity but rather circulating hormone concentrations following hepatic metabolism and gastrointestinal transit, which introduces a delay between any endocrine response and its measurable fecal output [25]. In reptiles, gastrointestinal transit time can be prolonged and highly variable, being influenced by diet and environmental conditions [26]. However, specific data on excretion delay times remain scarce for reptiles [26], hindering the characterization of the temporal relationship between HPI axis activity and fecal hormone output in the species studied here, and leaving open the possibility that transient endocrine responses to housing changes may have gone undetected.

Third, another potential explanation for the absence of significant variations between housing conditions could be a sustained activation of the HPA/HPI axis throughout the study, which might have masked any housing effect. However, in the absence of species-specific reference values for the four species studied, it is not possible to determine whether the FGM concentrations measured were elevated in absolute terms, as glucocorticoid profiles are species-specific and context-dependent [27]. Finally, in enriched terraria, animals had increased space (218.5 ± 82.6%; Mean ± SD), thus potentially increasing activity levels, which in some species, may result in elevated corticosterone production through activation of the HPA/HPI axis [1]. In racking systems, activity levels would likely be lower, but the lack of enrichment itself could act as a chronic stressor. This has been observed in green iguanas (*Iguana iguana*), where removing climbing structures led to an increase in fecal glucocorticoid metabolites [28].

**Figure 2:**
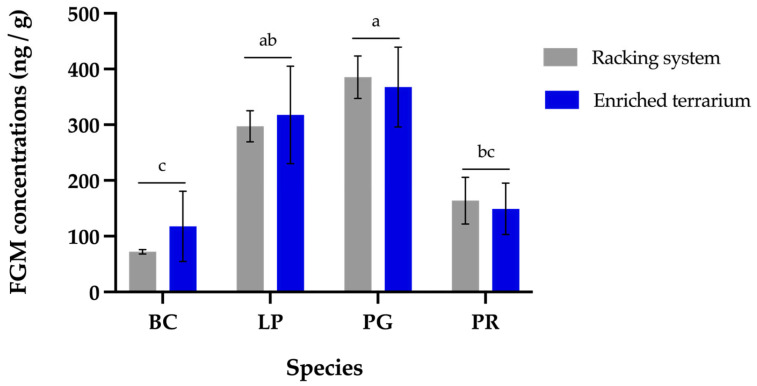
Mean (± SEM) fecal glucocorticoid metabolites (FGM) concentrations across four snake species: *Boa constrictor* (BC, n = 2), *Lampropeltis polyzona* (LP, n = 2), *Pantherophis guttatus* (PG, n = 4), and *Python regius* (PR, n = 4), under two housing conditions: racking system (gray) versus enriched terraria (blue). Letters (a, b, c) above these lines denote statistically significant differences among species (Tukey’s test, *p* < 0.05). Species not sharing a common letter exhibit significantly different FGM concentrations.

While housing conditions did not have a significant effect on FGM, snake species (the other fixed factor included the model) showed a significant effect (χ^2^ = 37.55; df = 3; *p* = 0.001). Tukey-adjusted pairwise contrasts indicated that PG had significantly higher FGM values than both BC (*p* = 0.001) and PR (*p* = 0.005). LP also showed higher FGM concentrations than BC (*p* = 0.026), as shown in Figure 2. These results suggest that PG and LP could have maintained higher metabolic and physiological activity during the study compared to PR and BC. This divergence aligns with their distinct ecological strategies. Active foraging species (such as PG and LP) require high baseline metabolic rates to sustain frequent movement and hunting. To support this energy mobilization, these species maintain higher baseline glucocorticoid levels [29,30]. Additionally, this elevated metabolic demand drives a more frequent feeding ecology and faster gut passage times, which explains the higher defecation frequency observed in the present study, with colubrid species accounting for approximately 75.9% of the total fecal samples collected (7.3 samples per individual on average, compared to 2.3 samples per individual in ambush predator species), as detailed in Table 1. In contrast, ambush predators such as BC and PR exhibit low basal metabolic rates and infrequent defecation, reflecting their inherent energy conservation strategies [29]. Consequently, the higher FGM concentrations and defecation frequencies observed in colubrid species likely indicate baseline energetic requirements and ecological strategies.

Finally, some limitations of the study must be acknowledged. First, the relatively small sample size of individuals per species and the heterogeneous sex and age representation in all the species could have restricted the statistical power and the generalization of the results. Secondly, the duration of each housing regimen was limited to 30 days. Although this represents a limitation of the study, the acute elevation of glucocorticoid levels due to the stress response to handling in reptiles has been shown to return to baseline concentrations within hours [31,32,33], and the integrative nature of fecal glucocorticoid metabolite measurements minimizes the potential confounding effect of transient acute stressors such as the initial transfer between enclosures performed in our study [34]. Thirdly, the variation in defecation frequency and gut passage time among individuals and species might have also introduced considerable variation masking the potential effects of changing the housing conditions as previously stated in reptiles [7]. Another important element of the present study is that snake feces had a large proportion of undigested prey material, such as fur, teeth, or bones, which could interfere with hormone extraction and quantification as previously described [12]. In the present study, the established sample processing protocol (Figure 1), which used sieving and careful removal of rodent fur with a slightly moistened brush, minimized this contamination.

## 4. Conclusions

The present pilot study provides preliminary evidence that fecal hormone analysis can detect physiological differences in glucocorticoid metabolite concentrations among captive snake species, supporting its potential as a non-invasive monitoring tool in reptiles. No significant effect of housing conditions on FGM concentrations was detected, although the small sample size and duration of each housing phase could have limited the interpretation of this result. Interspecific differences were observed, with active foraging species (PG and LP) showing higher FGM concentrations and defecation frequencies than ambush predator species (BC and PR), consistent with their distinct ecological strategies. Future studies should aim for larger and more balanced sample sizes, longer housing periods, and the establishment of species-specific baseline FGM values.

## Figures and Tables

**Table 1:** Descriptive summary of the individual snakes that participated in the study, including species, sex, age class, weight, and number of fecal samples collected per phase (P1, P2, P3) and volume (cm^3^) of the housing container (racking system vs. enriched terraria). P1 and P3 correspond to the racking system period. P2 corresponds to the enriched terraria period.

ID	Species	Sex	Age	Weight P1 (g)	Weight P2 (g)	Weight P3 (g)	Rack Volume (cm^3^)	Terrarium Volume (cm^3^)	n Feces P1	n Feces P2	n Feces P3	n Total Feces
1	*Boa constrictor*	F	Juvenile	671	642	619	15,964	63,070	0	1	1	2
2	*Boa constrictor*	F	Juvenile	530	515	532	15,964	63,070	1	1	1	3
3	*Lampropeltis polyzona*	M	Adult	323	328	356	15,964	62,370	3	1	3	7
4	*Lampropeltis polyzona*	M	Adult	339	337	323	15,964	37,098	5	4	1	10
5	*Pantherophis guttatus*	M	Juvenile	559	549	571	26,460	96,155	3	2	1	6
6	*Pantherophis guttatus*	F	Adult	434	422	429	15,964	58,752	5	1	2	8
7	*Pantherophis guttatus*	F	Adult	300	312	314	15,964	62,370	2	2	1	5
8	*Pantherophis guttatus*	F	Adult	349	359	343	15,964	37,098	4	2	2	8
9	*Python regius*	M	Adult	656	690	656	37,098	92,812	1	0	1	2
10	*Python regius*	F	Adult	715	686	742	15,964	60,260	0	1	0	1
11	*Python regius*	F	Adult	1730	1719	1814	63,070	109,032	1	2	0	3
12	*Python regius*	M	Adult	1537	1553	1483	63,070	159,831	2	1	0	3

## Data Availability

The raw data supporting the conclusions of this article will be made available by the authors on request.

## Abbreviations

The following abbreviations are used in this manuscript:

FGM	Fecal glucocorticoid metabolites
ACTH	Adrenocorticotropic hormone
HPI axis	Hypothalamic–pituitary–interrenal axis
EIA	Enzyme immunoassay
BC	*Boa constrictor*
LP	*Lampropeltis polyzona*
PG	*Pantherophis guttatus*
PR	*Python regius*
